# Seismic Discrimination between Earthquakes and Explosions Using Support Vector Machine

**DOI:** 10.3390/s20071879

**Published:** 2020-03-28

**Authors:** Sangkyeum Kim, Kyunghyun Lee, Kwanho You

**Affiliations:** Department of Electrical and Computer Engineering, Sungkyunkwan University, Suwon 16419, Korea; interpost94@skku.edu (S.K.); naman2001@skku.edu (K.L.)

**Keywords:** seismic discrimination, support vector machine, heterodyne laser interferometer, Hough transform, measurement accuracy

## Abstract

The discrimination between earthquakes and explosions is a serious issue in seismic signal analysis. This paper proposes a seismic discrimination method using support vector machine (SVM), wherein the amplitudes of the P-wave and the S-wave of the seismic signals are selected as feature vectors. Furthermore, to improve the seismic discrimination performance using a heterodyne laser interferometer for seismic wave detection, the Hough transform is applied as a compensation method for the periodic nonlinearity error caused by the frequency-mixing in the laser interferometric seismometer. In the testing procedure, different kernel functions of SVM are used to discriminate between earthquakes and explosions. The outstanding performance of a laser interferometer and Hough transform method for precision seismic measurement and nonlinearity error compensation is confirmed through some experiments using a linear vibration stage. In addition, the effectiveness of the proposed discrimination method using a heterodyne laser interferometer is verified through a receiver operating characteristic curve and other performance indices obtained from practical experiments.

## 1. Introduction

The discrimination between earthquakes and explosions is a serious issue in seismology. Seismometers at seismic stations record all types of earth vibrations in the region without the ability to clarify their origin. Considering that misidentified artificial seismic events, such as quarry blasts and underground nuclear tests, can lead to erroneous analyses, the classification of the signals’ source should be performed as a preliminary work prior to seismic signal processing and analysis [1]. General seismic discrimination is usually performed by visually inspecting the records of earthquakes and explosions or by calculating the characteristics of each record. However, this requires a great amount of work and time from earthquake analysts. Therefore, several earthquake discrimination errors occur. The seismic discrimination method using the machine learning classifier can shorten the workload and increase the reliability of classification results.

A considerable number of seismic signal discrimination methods based on statistical machine learning have been proposed [2,3,4,5,6,7]. Mousavi [2] developed a machine learning-based strategy to discriminate between deep microseismic events and shallow ones using logistic regression and artificial neural network models. The seismic features obtained from frequency and polarization attributes have higher correlation with seismic event’s source depth. Rabin [3] proposed a graph-based machine learning tool and the diffusion maps method for organizing a large number of events that are captured in a seismic station and for classifying new recorded events. Rabin also intended to apply kernel-based sensor fusion methods for extension of approach from single station to seismic network. Linville [4] suggested convolutional and recurrent neural network architectures on the task of binary event classification for tectonic earthquake and quarry blasts at local scales. The objective of this work was to build a model capable of reproducing analyst classifications on incoming data in near real time. Kuyuk [5] proposed an unsupervised learning-based self organizing map to distinguish micro-earthquakes from quarry blasts in the vicinity of Istanbul.

In this paper, the heterodyne laser interferometer [8] is used as a seismometer owing to its high precision, wide dynamic range, and nanometric resolution. For the laser interferometer to be used as a seismometer, the problem of the frequency cross-talk caused by imperfect optical components should be solved. This problem produces a nonlinearity error that restricts the measurement accuracy of the laser interferometer. Therefore, the Hough transform method, which is known as a line detection method, is applied to increase the measurement accuracy of the heterodyne laser interferometer. The precisely measured seismic data after the compensation of the nonlinearity error in laser interferometer is used as a test data for the discrimination of earthquake and explosion. Moreover, to discriminate between earthquakes and explosions, support vector machine (SVM) is applied as a machine learning model, and the amplitudes of the P-wave and the S-wave of the seismic signals are used to form the feature vectors, simplifying not only the early data processing procedure but also the computing process in building the SVM classifier. To increase the discrimination accuracy, SVM classifier is combined with the seismic data obtained from noncontact optical high-precision interferometer. In this study, the SVM-based discrimination method was executed using the limited seismic data measured from one station as a training dataset for the rapid response. In addition, as the dimensionality of the feature vectors is 2, the whole decision-making process of the discrimination system can be easily visualized, providing more convenience for follow-up analysis. The seismic data acquisition using a heterodyne laser interferometer as a seismometer enables discriminating between earthquakes and explosions. Owing to the wide dynamic range and high resolution, the microseismic waves measured by a laser interferometer can be used as a seismic data for SVM classifier. The amplitudes of the P-wave and the S-wave could be measured more precisely using Hough transformation-based nonlinearity error compensation in a laser interferometer. Moreover, the seismic events of earthquakes and explosions are realized using a linear stage.

This paper is organized as follows. In Section 2, the overall configuration of the heterodyne laser interferometer used as a seismometer is introduced, and the peak amplitude of the wave’s acceleration measured by the heterodyne laser interferometer is used to extract the feature vectors. Section 3 suggests the seismic discrimination method using the SVM function. The results of the seismic signal classification using different SVM kernel functions and the laser interferometer are shown in Section 4. In addition, the radial basis function (RBF) kernel functions and the laser interferometer-based seismic discrimination method demonstrate the improved discrimination accuracy. Finally, the conclusion is drawn in Section 5.

## 2. Hough Transform-Based Interferometric Seismometer Compensation

**Figure 1:**
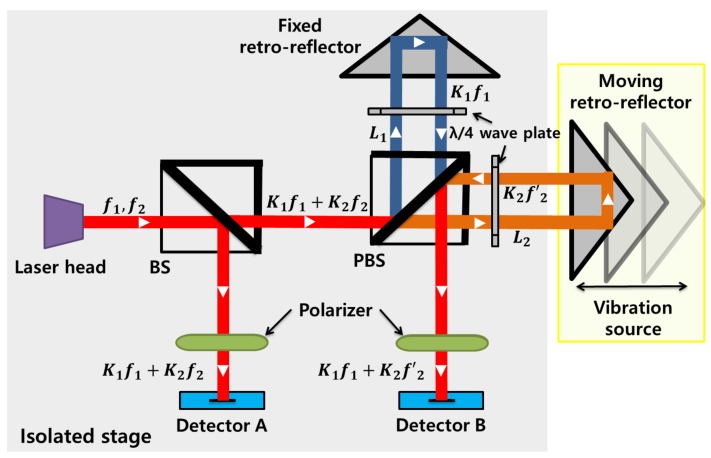
Heterodyne laser interferometer-based seismometer.

To discriminate the earthquake signals from those of explosions, the acceleration data are traced using the displacement variation measured by the heterodyne laser interferometer. Figure 1 shows the schematic diagram of the heterodyne laser interferometer-based seismometer. Two orthogonally polarized beams with two separate frequencies ($f_{1}$ and $f_{2}$) are emitted from a He-Ne laser head. Then, after passing a beam splitter (BS), the waves are equally split into two beams. The intensity of the reference beam is measured by the photodetector *A* as,

(1) $$\begin{array}{c} S_{A}\propto \frac{1}{2}\left(K_{1}^{2}+K_{2}^{2}\right)+K_{1}K_{2}\cos\left[2\pi \Delta ft+(\Phi_{2}-\Phi_{1})\right], \end{array}$$

where $K_{1}$ and $K_{2}$ are the magnitudes of the electric fields. $\Phi_{1}$ and $\Phi_{2}$ are the initial phases of the laser sources. Δf is the difference in frequency between $f_{1}$ and $f_{2}$. Then, the intensity of the measurement beam is observed by the photodetector *B* as,

(2) $$\begin{array}{ccc} S_{B} & \propto & \frac{1}{2}\left(K_{1}^{2}+K_{2}^{2}\right)+K_{1}K_{2}\cos\left[2\pi \Delta ft+(\Phi_{2}-\Phi_{1})+\Delta \Phi \right], \end{array}$$

where ΔΦ is the phase difference caused by the Doppler effect and $\Delta \Phi =2\pi (f_{2}^{\prime }-f_{2})t$. In addition, ΔΦ can be transformed into the displacement variation ΔL as,

(3) $$\begin{array}{c} \Delta \Phi =\frac{4\pi \zeta \Delta L}{\lambda }, \end{array}$$

where λ is the mean wavelength of $f_{1}$ and $f_{2}$, and ζ is the refractive index of air. With the use of the high pass filter, only the AC elements of $S_{A}$ and $S_{B}$ can be obtained, respectively. In addition, subsequent to passing through the lock-in amplifier, two orthogonal intensity signals are obtained as in [9,10]:

(4) $$\begin{array}{c} I_{x}\propto \frac{1}{2}K_{1}K_{2}\cos\left(\Delta \Phi \right),\;\;I_{y}\propto \frac{1}{2}K_{1}K_{2}\sin\left(\Delta \Phi \right). \end{array}$$

Using the inverse trigonometric function of $\tan^{-1}\left(I_{y}/I_{x}\right)$, the phase value ΔΦ can be represented as in Equation (3).

Nonlinearity is introduced when laser beams pass through the PBS. In an ideal case, the two orthogonal beams are perfectly separated by the PBS. Then, one beam source of frequency $f_{1}$ would move to the fixed retro-reflector, and the other orthogonal beam of frequency $f_{2}$ would move toward the moving retro-reflector. However, in practical measurements, imperfect polarization in the PBS generates frequency mixing. The beam sources that proceed to the retro-reflectors are contaminated by each other. Therefore, the intensity of the measurement signal collected in photodetector *B* includes the nonlinearity error components [11,12,13]. Thus, the intensity of the measurement signal with the nonlinearity from the photodetector *B* can be represented as follows:

(5) $$\begin{array}{ccc} S_{B,N} & \propto & \frac{1}{2}\left(K_{1}^{2}+K_{2}^{2}+\alpha^{2}+\beta^{2}\right)+K_{1}K_{2}\cos\left[2\pi \Delta ft+\Delta \Phi +(\Phi_{2}-\Phi_{1})\right]\\ & & +K_{1}\beta \cos\left[2\pi \Delta ft+(\Phi_{\beta }-\Phi_{1})\right]+K_{2}\alpha \cos\left[2\pi \Delta ft+(\Phi_{2}-\Phi_{\alpha })\right]\\ & & +K_{1}\alpha \cos\left[\Delta \Phi +(\Phi_{\alpha }-\Phi_{1})\right]+K_{2}\beta \cos\left[\Delta \Phi +(\Phi_{2}-\Phi_{\beta })\right]\\ & & +\alpha \beta \cos\left[2\pi \Delta ft-\Delta \Phi +(\Phi_{\beta }-\Phi_{\alpha })\right], \end{array}$$

where α and β denote the magnitudes of frequency cross-talk components caused by nonorthogonal source beams, inaccurate alignment, and reflection paths through the PBS. The results of ${\hat{I}}_{x}$ and ${\hat{I}}_{y}$, including the nonlinear errors, can be expressed as follows.

(6) $$\begin{array}{ccc} {\hat{I}}_{x} & \propto & \frac{1}{2}\left(K_{1}K_{2}+\alpha \beta \right)\cos\Delta \Phi +\frac{1}{2}\left(K_{1}\beta +K_{2}\alpha \right),\\ {\hat{I}}_{y} & \propto & \frac{1}{2}\left(K_{1}K_{2}-\alpha \beta \right)\sin\Delta \Phi . \end{array}$$

The Hough transform [14,15,16] is an algorithm that is frequently used to find contour lines in image processing. The basic idea of Hough transformation is to find the intersection point among the line equations that comprise the slopes and *y*-intercepts of the given data. However, it is difficult to accumulate data using slopes and intercepts in the *x*–*y* coordinate system, as the slopes in this system range from zero to infinity. To overcome this problem, all the lines passing through one particular data point are represented as polar coordinates in the rθ-plane. Then, to find the intersection point, the rθ-plane is divided into small cells similar to those of a map. After transforming all the data into the rθ-plane, the cell in which the coordinates accumulate most is selected as the intersection point. For the nonlinearity error compensation using the Hough transform method, the relations between the reference signal ($I_{x}$, $I_{y}$) and the measurement signal (${\hat{I}}_{x}$, ${\hat{I}}_{y}$) are transformed into the rθ-plane as ($r_{x}$, $\theta_{x}$) and ($r_{y}$, $\theta_{y}$), which have the following relations:

(7) $$\begin{array}{ccc} r_{x} & = & I_{x}\cos\theta_{x}+{\hat{I}}_{x}\sin\theta_{x},\\ r_{y} & = & I_{y}\cos\theta_{y}+{\hat{I}}_{y}\sin\theta_{y}, \end{array}$$

where $r_{x}$ and $r_{y}$ are the distances from the origin to the points ($I_{x}$, ${\hat{I}}_{x}$) and ($I_{y}$, ${\hat{I}}_{y}$), respectively, and $\theta_{x}$ and $\theta_{y}$ are the angles of ($I_{x}$, ${\hat{I}}_{x}$) and ($I_{y}$, ${\hat{I}}_{y}$), respectively. To apply the Hough transform method in the laser interferometer system, the ranges of $r_{x,y}$ and $\theta_{x,y}$ need to be limited. Thus, the range of $r_{x,y}$ is set as $0\leq r_{x,y}\leq \sqrt{2}$ because the maximum values of $I_{x}$ and ${\hat{I}}_{x}$ are 1. In addition, because of the periodicity, the range $0\leq \theta_{x,y}\leq \pi$ can be used without the loss of generality. Then, an accumulator is utilized based on $r_{x,y}$ and $\theta_{x,y}$ in the Hough space for the compensation process. After the projection into the Hough space, the Hough accumulator counts the number of projections per each cell. The size of the Hough accumulator is m×n in two dimensions. As the values of *m* and *n* increase, the relationship between the reference and the measurement signals can be more accurately determined.

**Figure 2:**
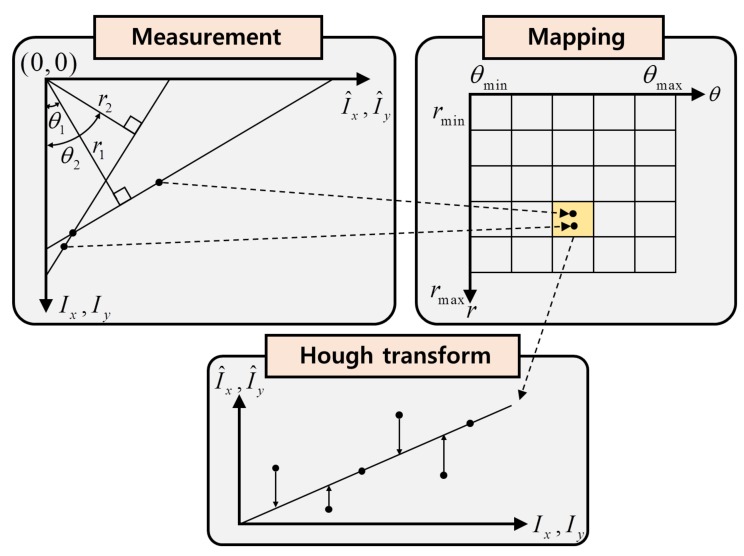
Application of the Hough transform in laser interferometer.

All the datasets of (r, θ) obtained from the Hough transform are projected into two-dimensional accumulator cells. This process is shown in Figure 2, where the cell ($\bar{r}$, $\bar{\theta }$) that has the maximum projection number is selected as the optimal solution. In the Hough transform method, the cell selected in the rθ-plane represents the relation between the reference and the measurement signal. In addition, the optimal solutions for the distance $r_{x,y}$ and the angle $\theta_{x,y}$ are taken as an average of the multiple values from the selected cell, which can be expressed as follows:

(8) $$\begin{array}{c} \bar{r}=\frac{1}{n}\sum_{i=1}^{n}r_{i},\;\;\bar{\theta }=\frac{1}{n}\sum_{i=1}^{n}\theta_{i},\;\;\;i=1,\cdots ,n, \end{array}$$

where *n* is the number of data points that fell in the selected cell. By using the $\bar{r}$ and $\bar{\theta }$ obtained from the Hough transform method, we can find the optimal solution for $I_{x}^{*}$ and $I_{y}^{*}$ as follows.

(9) $$\begin{array}{c} I_{k}^{*}=-\frac{\sin{\bar{\theta }}_{k}}{\cos{\bar{\theta }}_{k}}{\hat{I}}_{k}+\frac{{\bar{r}}_{k}}{\cos{\bar{\theta }}_{k}},\;\;\;k=x,y. \end{array}$$

## 3. Seismic Signal Discrimination Using SVM

In seismology, the amplitudes of the the P-wave ($A_{p}$) and the S-wave ($A_{s}$) are defined as the peak values of the P-wave and the S-wave, respectively. Many studies have shown that the ratio between $A_{p}$ and $A_{s}$ is an effective indicator to discriminate between earthquakes and explosions data with distances from 50 to 200 km [17,18,19,20,21]. In many cases, earthquakes show that $A_{s}$ is bigger than or equal to $A_{p}$, whereas explosions show that $A_{p}$ is bigger than $A_{s}$.

**Figure 3:**
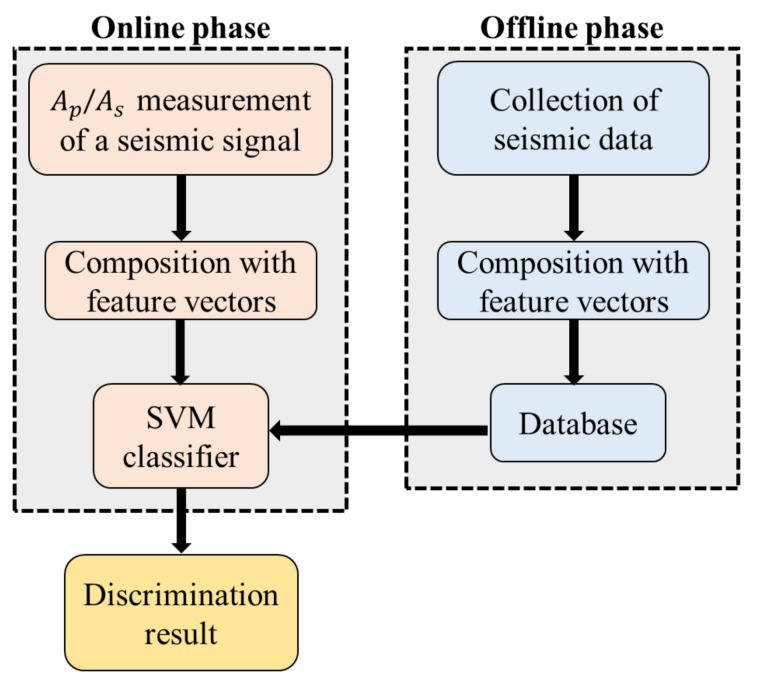
Framework of seismic discrimination algorithm.

In this paper, the seismic discriminating method is processed in two phases as shown in Figure 3. In the offline phase, which can also be called the training phase, a database is built to contain the information of $A_{p}$ and $A_{s}$ together with the label (earthquake or explosion) of the separately-collected seismic signal. Then, SVM classifier is trained by these data, and the decision function of the SVM classifier is fixed. In the online phase (the testing phase), we input the values of $A_{p}$ and $A_{s}$ of the seismic signal into the SVM classifier, and the output is the discrimination result of the seismic signal.

The SVM is a supervised and non-probabilistic learning model that is popular and effective [22,23,24]. In the offline phase, the set of training data can be expressed as $\left\{\left(x_{i},y_{i}\right)\right\}$, $x_{i}\in R^{2}$, $y_{i}\in \left\{+1,-1\right\}$, i∈{1,2,⋯,k}, where $x_{i}$ is a two-dimensional feature vector with the value of the amplitudes ($A_{p}$ and $A_{s}$) of the P-wave and the S-wave of a seismic signal; $y_{i}$ is a label, with +1 representing an earthquake and −1 representing an explosion; and *k* is the number of the training data. As a hyperplane of the SVM can be written as

(10) $$\begin{array}{c} wx_{i}+b=0, \end{array}$$

then the decision function of the SVM is

(11) $$\begin{array}{c} f\left(x\right)=\mathrm{sgn}\left(wx_{i}+b\right), \end{array}$$

where *w* is the weight vector and *b* is the unregularized bias term. To maximize the margin between the two classes of data, as shown in Equation (12),

(12) $$\begin{array}{cc} {\mathrm{maximize}}_{w,b} & \frac{2}{∥w∥},\\ \mathrm{subject}\;\mathrm{to} & y_{i}\left(wx_{i}+b\right)\geq 1, \end{array}$$

Lagrange multipliers method with Karush–Kuhn–Tucker (KKT) conditions [25,26] are then applied. As maximizing the margin value 2/∥w∥ is equivalent to minimizing ${∥w∥}^{2}/2$, we transform the objective function into a minimum form, and the Lagrange function is represented in Equation (13),

(13) $$\begin{array}{c} L\left(w,b,\mu \right)=\frac{{∥w∥}^{2}}{2}-\sum_{i=1}^{n}\mu_{i}\left[y_{i}\left(wx_{i}+b\right)-1\right], \end{array}$$

where $\mu_{i}$ is the Lagrange multiplier, which is nonzero for the support vectors. According to the complementary slackness in KKT conditions,

(14) $$\begin{array}{c} \mu_{i}\left[y_{i}\left(wx_{i}+b\right)-1\right]=0. \end{array}$$

By taking the partial derivative of the Lagrange function of Equation (13) with respect to *w* and according to the KKT conditions, we obtain

(15) $$\begin{array}{c} w=\sum_{i=1}^{n}\mu_{i}y_{i}x_{i}. \end{array}$$

Therefore, the decision function of the SVM can be transformed to

(16) $$\begin{array}{c} f\left(x\right)=\mathrm{sgn}\left\{\sum_{i=1}^{n}\mu_{i}y_{i}\left(x_{i}\cdot x\right)+b\right\}. \end{array}$$

Considering that the seismic signal discrimination problem is a linear non-separable case, the kernel functions $K\left(x_{i},x_{j}\right)=\psi \left(x_{i}\right)\cdot \psi \left(x_{j}\right)$ are used to map the original input vector space to a high dimensional feature space where an optimal hyperplane can be found [27].

## 4. Simulation and Experiment for Seismic Event Discrimination

**Figure 4:**
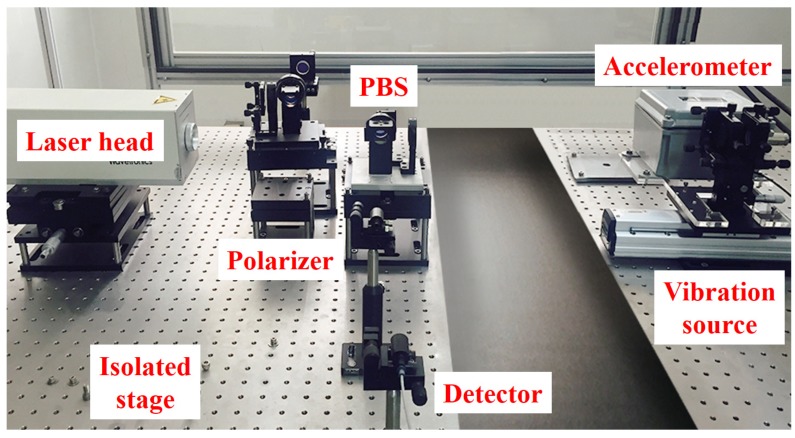
Seismic wave measurement using a laser interferometer.

In this section, the performance of the seismic event discrimination method using a SVM classifier and a heterodyne laser interferometer is verified. As a precision seismometer, the laser interferometer (Wavetronics: WT-307B) was used. The high performance of the laser interferometer-based precision seismometer can be confirmed in [10]. The laser interferometer is installed on the optical table (EKSMA Optics: 778-5060) to isolate the system from the vibration of a seismic wave. The linear motion stage (Sciencetown: PSA6520) is activated by a two-phase stepping motor for the seismic event realization. Figure 4 shows the seismic wave measurement system. In the experiment, the amplitudes ($K_{1}$ and $K_{2}$) of the laser signal, the air refractive index (ζ), and the two-frequency beams ($f_{1}$ and $f_{2}$) in Equation (3) were 1 V, 1.00000002665, $\lambda_{1}$ = 632.9912576 nm, and $\lambda_{2}$ = 632.9912604 nm, respectively.

**Figure 5:**
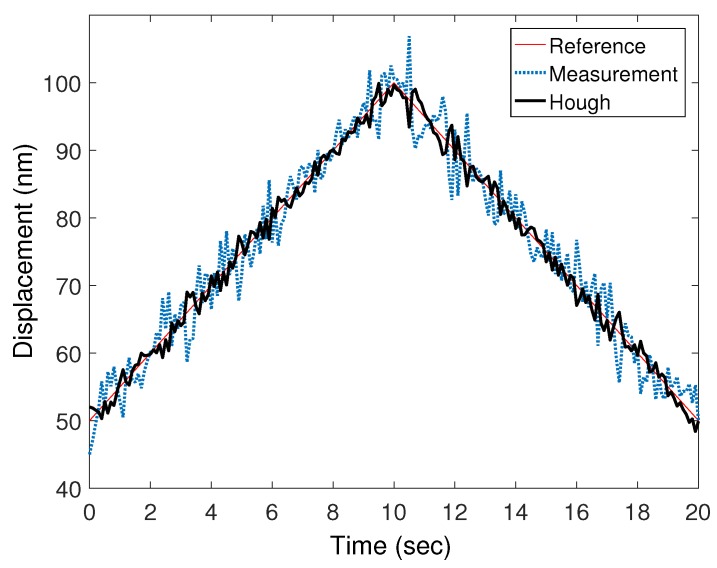
Nonlinearity error compensation by Hough transform method.

Using the Hough transform method proposed in Section 2, the nonlinearity error was compensated to increase the accuracy of the seismic signal measurement. Figure 5 shows a simulation result of the nonlinearity error compensation by the Hough transform method for a linear stage that moves following a triangular input command. In the figure, the thin dotted line is an uncompensated laser interferometer signal, the thin solid line is a reference signal, and the thick solid line represents the compensated signal. The compensation result was closer to the reference signal than to the uncompensated signal, thereby validating the ability of the Hough transform compensation algorithm in reducing the nonlinearity error. Therefore, the amplitudes of P-wave and S-wave can be measured precisely through Hough transformation-based laser interferometric seismometer. To demonstrate the performance of our proposed method, the simulation and experiment process were performed following the three procedures. As a first step, we obtained the seismic dataset recorded by Sitting Bull Academy station in Apple Valley, California. The dataset was measured and discriminated in 2017 by the United States Geological Survey (USGS) and the Incorporated Research Institutions for Seismology (IRIS). The seismic dataset was used as training data for the configuration of SVM classifiers. Unusual seismic events such as ultra-low frequency earthquakes were excluded as training data. As a second step, the other seismic test dataset was selected in the same way. The acceleration information of the selected dataset was used as an input to a linear stage for data reconstruction. The seismic dataset reconstructed by a linear stage was measured by the Hough transform-based heterodyne laser interferometer. As a third step, to discriminate the seismic data which were precisely measured by the Hough transform-based laser interferometer, the SVM classifier was applied to test data.

**Figure 6:**
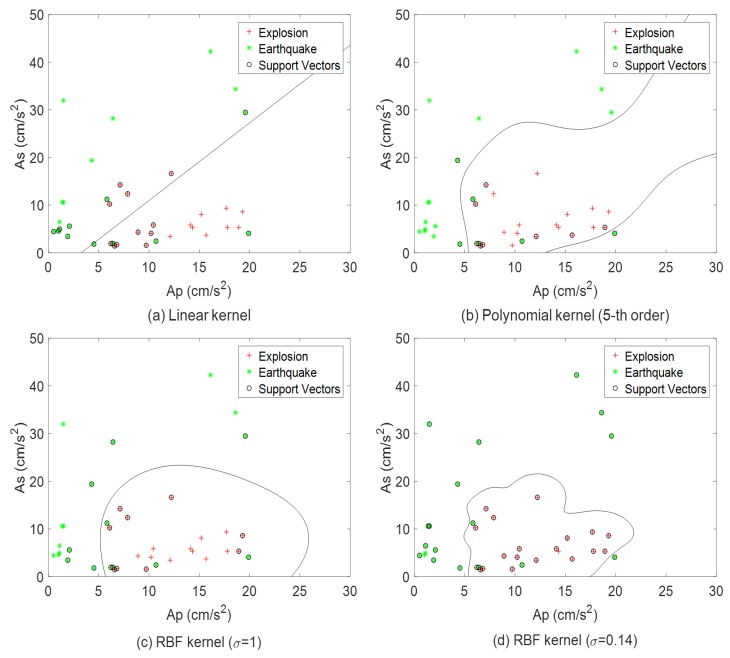
SVM classifier with different kernel functions.

For the simulation of the seismic discrimination, 20 earthquake and 20 explosion seismic data with distances from 50 to 200 km were used to train the SVM classifiers. Both earthquake and explosion seismic data were measured by the same station under the identical condition of geological characteristics and measurement environment. By choosing different kernel functions, various SVM classifiers can be built according to the same training data. As shown in Figure 6, the linear kernel $K\left(x_{i},x_{j}\right)=x_{i}\cdot x_{j}$, the fifth polynomial kernel $K\left(x_{i},x_{j}\right)={\left(\left(x_{i}\cdot x_{j}\right)+1\right)}^{5}$, and the RBF kernel $K\left(x_{i},x_{j}\right)=\exp(-∥x_{i}-x_{j}{∥}^{2}/\sigma )$, (σ=1,0.14) were used to train the SVM, respectively. The *x*- and *y*-axis are the amplitudes of the P-wave and the S-wave, respectively. The symbols of the asterisks represent the earthquake data, and the cross symbols represent the explosion data, respectively. In addition, the support vectors that determine the hyperplane are marked with circles. The selection of the parameter (σ) in RBF kernel function is important since the decision of σ affects the discrimination accuracy and causes the overfitting problem. In this study, we obtained the optimal parameter (σ=0.14) through some simulations with different σ values. As shown in Figure 6, the outstanding discrimination performance of SVM with RBF kernel (σ=0.14) is confirmed.

**Figure 7:**
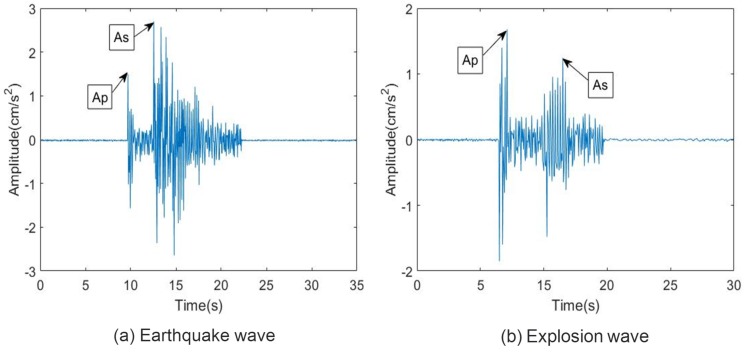
Amplitudes of P-wave and S-wave using laser interferometer.

**Figure 8:**
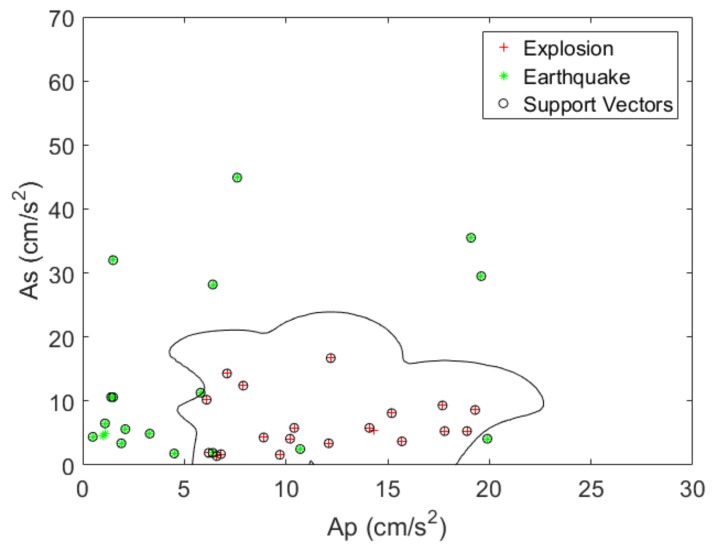
SVM classifier with RBF kernel function (σ=0.14) using laser interferometer.

To prove the performance of the SVM classifier, the seismic signal that was observed by the USGS and IRIS was reconstructed by the linear stage and measured by the heterodyne laser interferometer. Then, to verify the accuracy of the seismic wave measurements, an accelerometer (Mitutoyo: JEP-8A3) was mounted on the linear stage. The frequency response, measurement range, dynamic range, and sensitivity of JEP-8A3 were 0–400 Hz, ±3000 Gal, 145 dB, and 0.306 V/(m/s${}^{2}$) ±3%, respectively. Figure 7 shows an example of $A_{p}$ and $A_{s}$ of a reconstructed seismic signal. The linear stage was activated to artificially simulate the earthquake and explosion data, and the seismic signals generated by the linear stage were measured by the laser interferometer. The precisely measured seismic data, of which the nonlinearity error was compensated through Hough transform method, were used as a test seismic dataset to discriminate between earthquake and explosion. Then, to determine the new seismic signal reconstructed by laser interferometer, we classified the seismic signal using SVM, which was trained by 40 seismic data from USGS. Moreover, the seismic magnitude of training dataset was restricted between 1.8 and 3.0. The SVM classifier using a RBF kernel function (σ=0.14) was applied to 40 new datasets. The test dataset has the same condition of magnitude 1.8 < *M* < 3.0 with training data. Figure 8 shows the classification results of the reconstructed dataset with the linear stage and the laser interferometer.

**Figure 9:**
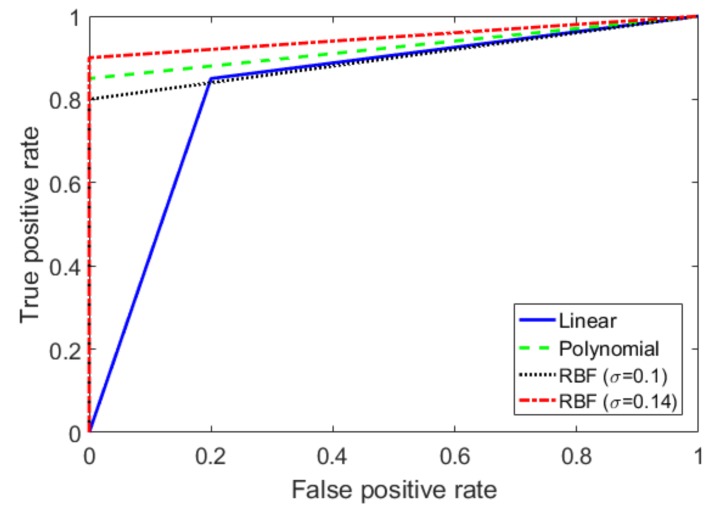
ROC curve comparison for different SVM classifiers.

The receiver operating characteristic curve (ROC) and the area under the curve (AUC) were adopted as effective measures of discrimination accuracy. The ROC curve is a graphical analysis tool that was initially proposed in the field of the signal detection to select the optimal detecting model or the detecting threshold of the optimum model [28]. The process of plotting the ROC curve is to calculate the true positive rate (TPR) and the false positive rate (FPR) as the threshold of the classifier is changed. The TPR is equal to the ratio of the true positive (TP) correctly classified by the system to the sum of the TP and the false negative (FN) incorrectly classified by the system. TPR is obtained as TPR=TP/(TP+FN). Likewise, FPR is defined as the ratio of the false positive (FP) to the sum of the FP and the true negative (TN), FPR=FP/(FP+TN). When the threshold increases, it becomes more difficult for the data to be classified as the positive class, leading to a simultaneous decrease in the values of the TPR and FPR [29]. For an ideal classifier, the ROC curve should cross the coordinate (0, 1) when an optimal threshold value is set, which means that all the positive data were correctly identified and no negative data were classified as a positive class. According to the principle of the ROC curve, the AUC can be used to evaluate the performance of the classifiers. While an ideal classifier has an AUC value of 1, the AUC values of our tested SVM classifiers with the linear kernel, the fifth-order polynomial kernel, the RBF kernel with a σ of 1, and the RBF kernel with a σ of 0.14 are 0.83, 0.93, 0.90, and 0.95, respectively, as shown in Figure 9.

Then, to verify the performance of the SVM models, the evaluation indicators of the machine learning classifier were applied in the form of a confusion matrix [30]. In Table 1, the precision (TP/TP+FP), recall (TP/TP+FN), and AUC values of the tested machine learning models are listed. According to Table 1, the SVM classifier using the RBF kernel function with σ=0.14 performs best in the test.

**Figure 10:**
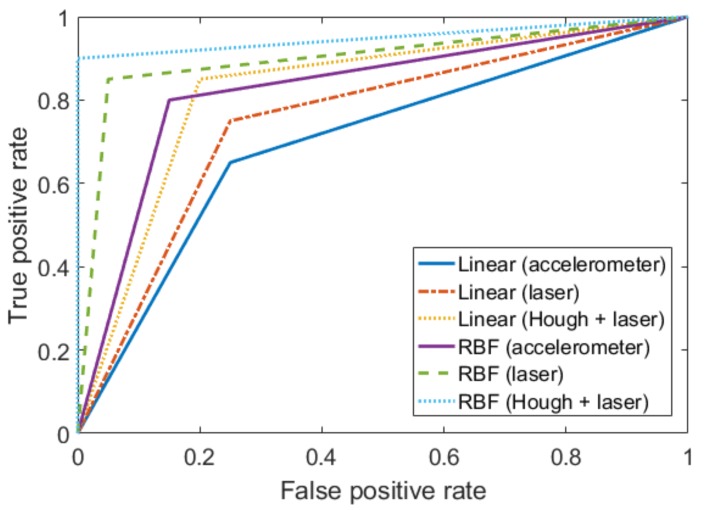
ROC curve comparison for different test datasets.

Moreover, we confirmed the performance of our proposed discrimination method by comparing the results of SVM classifiers for different test datasets as shown in Figure 10 and Table 2. In Figure 10, the solid lines denote the ROC curves of linear and RBF (σ=0.14) kernel-based SVM classifiers using the accelerometer measurement data, respectively. The dashed and dotted lines represent the ROC curves of SVM classifiers using seismic datasets of which the nonlinearity error is compensated and uncompensated, respectively. As shown in Figure 10 and Table 2, the RBF kernel-based SVM classifier denotes the better performance compared to linear kernel-based classifier in all test datasets. Moreover, the discrimination using seismic data compensated by Hough transform shows outstanding performance in both linear and RBF kernel-based SVM classifications.

## 5. Conclusions

In this paper, a SVM-based seismic discrimination method is proposed by using the amplitudes of the P-wave and the S-wave as the feature vectors. A heterodyne laser interferometer was used to obtain precise data of the seismic wave. Hough transformation method was applied to compensate for the nonlinearity error of the measurements and to obtain accurate feature vectors from the body wave. Moreover, the decision function of the SVM classifier was set by the collected past data and the selected kernel function. As a new seismic signal is collected, earthquake discrimination can be executed using the decision function of the SVM with the additional input data. As a result, seismic discrimination using SVM and the laser interferometer has shown a high discrimination accuracy. The improved performance of the seismic signal measurement using Hough transform-based laser interferometer was proved with simulation results. Moreover, the effectiveness of our proposed discrimination algorithm was confirmed through the results of ROC and AUC.

## Figures and Tables

**Table 1:** Performance evaluation of SVM models.

Machine Learning Model	Precision	Recall	AUC
Linear kernel SVM	0.78	0.85	0.83
5-th order polynomial SVM	1.00	0.85	0.93
RBF kernel SVM (σ=1)	1.00	0.80	0.90
RBF kernel SVM (σ=0.14)	1.00	0.90	0.95

**Table 2:** Performance evaluation of SVM models for different test datasets.

Measurement Method	Precision	Recall	AUC
Linear (accelerometer)	0.81	0.65	0.70
Linear (laser interferometer)	0.75	0.75	0.75
Linear (laser interferometer+Hough)	0.78	0.85	0.83
RBF with σ=0.14 (accelerometer)	0.86	0.80	0.83
RBF with σ=0.14 (laser interferometer)	0.96	0.85	0.90
RBF with σ=0.14 (laser interferometer+Hough)	1.00	0.90	0.95
